# On Bleeding in Peripneumony

**Published:** 1816-02

**Authors:** Robert Kinglake


*For the London Medical and Physical Journal.*

### *On Bleeding in Peripneumony;* by Dr. Robert Kinglake.

FACTS have lately occurred that fully confirm my previous opinion, that bleeding is not usually carried far enough in the early stage of this threatening malady, either to insure early recovery, or to obviate the production of irreparable mischief. Bleeding, in this disease, as in most others of an inflammatory nature, is usually directed more against the violence of the attending symptoms, than against the actual morbid state itself. The necessary consequence of this insufficient remedy is a mitigated continuance of the disease, and nothing like its complete extinction. The partial benefit thus obtained often proves injuriously fallacious, by inducing an erroneous calculation as to the event, and a premature remission or neglect of necessary remedies.

An inflammatory affection is very far from being subdued when repressed in its violence. The highly-excited state of vital power constituting inflammatory disease cannot be promptly and effectually reduced within safe and convalescent limits, without extending the remedy employed to the length of radically correcting the altered conditions on which the disease essentially depends. What, then, in inflammatory excitement, are those morbidly altered conditions? They consist in an excess of vital action that at once threatens a destructive exhaustion of living power, or such a disorganization of structure as would be incompatible with animal life. If, then, the immediate excitement be the efficient cause of this dangerous state of disease, it must be admitted that the most direct and effectual mode of relief should be firmly and unsparingly employed. No article in the materia medica can be implicitly depended on for its powers in overcoming this disease with sufficient rapidity to prevent the irreparable injury which its undue protraction might occasion. No medicinal influence can quickly enough arrest the hurtful progress of inflammatory action incessantly augmenting under the unabated stimulus of vascular distention. No exciting power whatever is so universal, and so important in its operation in the animal economy, as that of the stimulus of distention. Both life and health are essentially connected with its natural state, and its excessive action may be regarded as the proximate cause of inflammatory disease. The obvious remedy, then, for this species of disease is adequate bleeding. The existing circumstances must determine to what length it should be carried to accomplish the curative object in view. The state of the pulse, and the aspect of the blood, blood, are, in general, sufficient criteria to direct the judgment in that point; but the paramount and infallible guide in those cases is the degree or quantum of disease to be subdued.

It should be recollected that the complete cure, and not the temporary relief only, is to be attempted. For this purpose, the sanguiferous vessels should be durably replenished as far as may be necessary to overcome the inflammatory action. This can only be effected by reducing the powers of life to an ebb that would be too low to admit of a continuance of inflammatory action. Scanty and desultory bleedings will not answer this end: they must be ample, and renewed at intervals sufficiently short to preserve and carry on the advantage of a former bleeding. On this scheme of making one bleeding subservient to another, in securing the curative benefit of vascular depletion, it has been for some time past my practice to direct an emission of blood in high inflammatory diseases, such as peripneumony, phrenitis, hepatitis, enteritis, &c. every six hours, until the characteristic symptoms of the affection shall disappear, or the pulse shall have wholly lost its hardness, and have evidently declined in strength.

In many instances this practice has been pursued with the most happy and encouraging success, under circumstances in which the usual treatment would have probably been unavailing. The practice of drawing blood once in either twenty-four or forty-eight hours in acute inflammatory affections has the effect only of diminishing the apparent violence of symptoms, whilst the exhausting and disorganizing influence of protracted inflammation is doing irretrievable injury. An inflammatory disease unchecked, or rather not cured in the early stage of its existence, will, after the lapse of a week or ten days, have induced a perilous state of indirect debility, that would render the free bleeding applicable to inflammatory action at its commencement wholly inadmissible.

Many cases of this description are within my professional recollection, in which the opportunity of benefiting by early bleeding was unhappily lost, and could not be supplied in the late stage of the disease by any thing better than inadequate and doubtful means of assistance. Much observation has enabled me to come to this practical conclusion, that three bleedings, to the extent of sixteen ounces at each time, performed at equal distances within twenty-four hours, will go farther towards overcoming visceral inflammation than twice that quantity would do in double that period, and with infinitely infinitely less risk of either gangrenous exhaustion, or destructive disorganization.

The guide for the precise time of renewing a bleeding in inflammatory disease should be at the *incipient* stage of the *re-action* ensuing the vascular contraction that takes place after losing a considerable quantity of blood. If this re-action be permitted to go on and establish itself, the former violence of the inflammatory disease, of which it is a part, will be restored, and thus may the conflict be prolonged to an indefinite duration, and with consequences full of danger to the safety of the patient.

Thus it may be clearly seen that bleedings may, in some instances, be renewed even at intervals of three hours instead of six, provided the vascular re-action referred to should have so soon shown itself. It would be unwarrantable, and necessarily hazardous, to repeat bleeding before some degree of re-action had occurred, as it might incapacitate the resources of life from making head against the debilitating influence of the former bleeding, in which case life itself even might be extinguished. This is illustrated by the destructive effects that have been known to result from premature bleedings in concussions of the brain. The paralizing influence of a violent shock abruptly inflicted on the nervous system must subside on these occasions, and returning energy be manifested by re-active efforts in the vascular system, before bleeding could be profitably or safely employed in such circumstances.

The large and frequently renewed bleedings here proposed must be understood to be exclusively confined to the early stage of acute inflammatory diseases. The object is to cut short a morbid violence that could not be prolonged without imminent risk, and which, therefore, should not be tampered with, lest an undue forbearance of the necessary means of relief should, as I verily believe to be often the case, baffle every subsequent endeavour to avert the destructive consequences. In the more advanced and chronic states of inflammatory disease, small bleedings, at longer distances, may importantly benefit under occasional circumstances of vascular plethora connected with visceral congestions and other forms of disease; but on this subject I shall reserve what further occurs to me to say for a future communication.

*Taunton;*

*January* 6, 1816.

